# Postpartum blood–milk barrier integrity in Holstein dairy cows with contrasting metabolic status

**DOI:** 10.3168/jdsc.2026-1104

**Published:** 2026-08-24

**Authors:** Tobias Heiri, Julia Eichinger, Jonas Hendricks, Anna-Maria Reiche, Frigga Dohme-Meier, Josef J. Gross

**Affiliations:** 1 Veterinary Physiology, Vetsuisse Faculty, University of Bern, CH-3012 Bern, Switzerland; 2 Ruminant Nutrition and Emissions, Agroscope, CH-1725 Posieux, Switzerland

## Abstract

The transition from colostrum to mature milk secretion depends on progressive closure of the blood–milk barrier. An increased metabolic load around calving is associated with inflammatory mediators known to impair tight junction integrity in other epithelial tissues, but whether it alters blood–milk barrier closure after parturition is unclear. This study investigated changes in blood–milk barrier permeability markers in dairy cows under different metabolic loads after calving. Twenty multiparous Holstein cows were assigned to either a high- or a low-energy feeding regimen during the dry period, resulting in experimental groups with either greater (HC; 3.77 ± 0.12) or lower (LC; 2.72 ± 0.12) BCS at calving. Postpartum, the HC group received a reduced and the LC group a standard level of energy-rich concentrate to further enhance metabolic divergence. The HC cows consequently had a more negative energy balance and greater nonesterified fatty acid and BHB concentrations than LC cows, indicating greater metabolic load at the onset of lactation. Blood–milk barrier integrity was assessed during the first 5 d of lactation using protein-based markers (albumin, lactate dehydrogenase), milk electrolytes (sodium, potassium, chloride), and SCC. Protein-based permeability markers and SCC showed no differences. However, milk Na and K concentrations and the Na/K ratio indicated transiently greater epithelial permeability in HC cows during the first 2 milkings, whereas a transient Cl group difference at the second milking did not translate into an overall effect. These findings suggest that the postpartum closure of the blood–milk barrier is largely unaffected by metabolic stress, although subtle and transient permeability differences detectable by electrolyte markers cannot be excluded.

## Graphical Abstract

**Figure:**
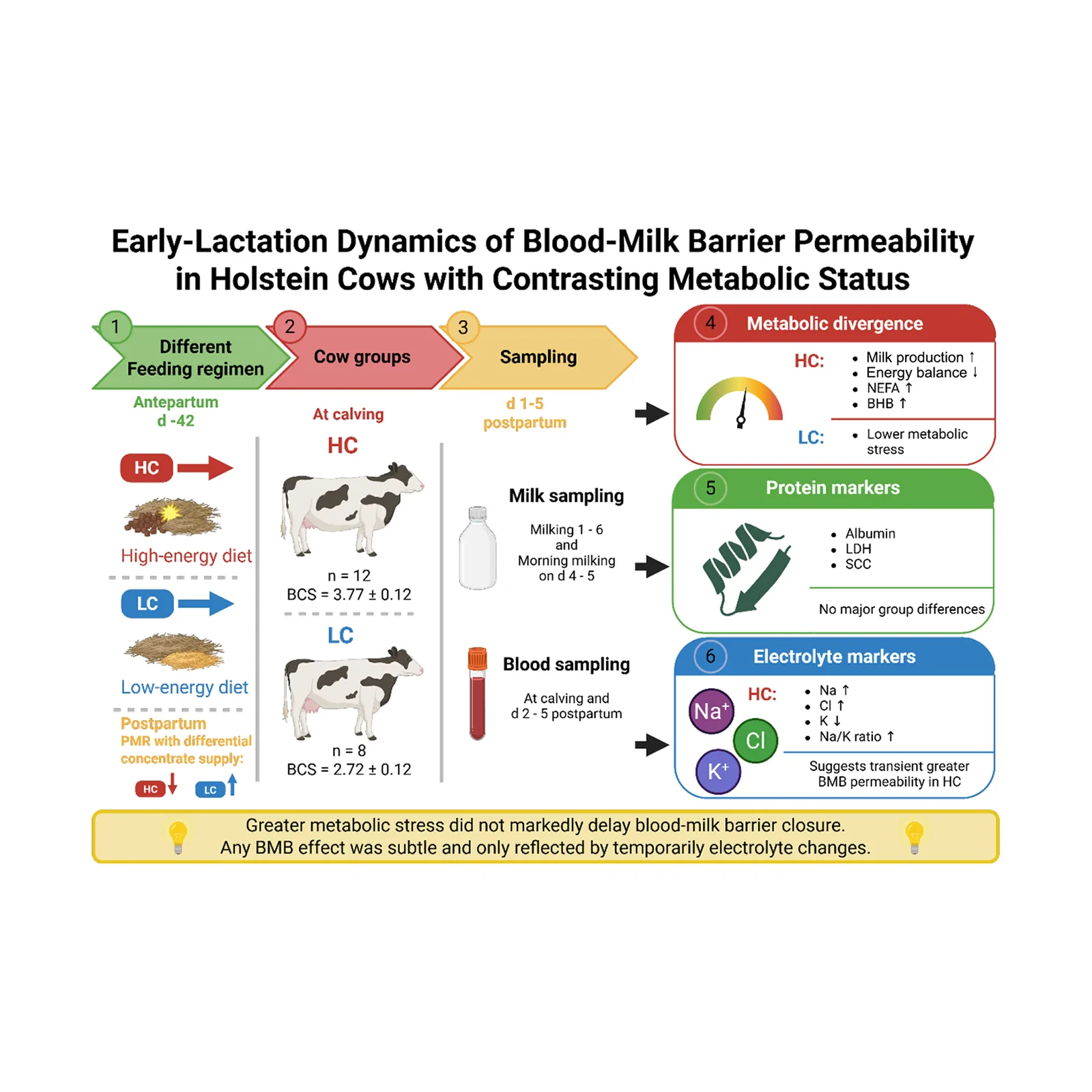

The transition from the dry period to lactation involves functional remodeling of the mammary gland (Stelwagen, 2025) and a subsequent shift from colostrum to transition milk secretion (Tsioulpas et al., 2007). Compared with mature milk, colostrum is rich in immunoglobulins, immune cells, growth factors, and other blood-derived components essential for neonatal survival and early development (Lopez and Heinrichs, 2022). Colostrogenesis depends on high paracellular permeability of the mammary epithelium, enabling nonselective transfer of blood components between adjacent mammary epithelial cells, in addition to active transcellular transport mechanisms for specific proteins, such as IgG1, via the FcRn pathway (Baumrucker et al., 2021). During the first days postpartum (**p.p.**), the progressive closure of mammary epithelial tight junctions (**TJ**) as part of the blood–milk barrier (**BMB**) restricts solute movement between blood and milk (Stelwagen, 2025). This process drives the transition from colostrum toward mature milk secretion (Nguyen and Neville, 1998; Stelwagen and Singh, 2014). Regulation of TJ integrity at the onset of lactation is complex and influenced by numerous endocrine, metabolic, and inflammatory signals (Wellnitz and Bruckmaier, 2021; Stelwagen, 2025). Integrity of the BMB is typically inferred indirectly from permeability markers such as blood-derived proteins in milk, leakage of milk components into blood, and changes in milk electrolyte concentrations (Stelwagen et al., 1995; Kessler et al., 2019). Cows with high prepartum BCS are more likely to experience severe negative energy balance (**NEB**) after calving and, consequently, a greater metabolic load (Roche et al., 2009). Reflected by increased plasma nonesterified fatty acids (**NEFA**) and BHB concentrations (Cruz et al., 2024; Toralová et al., 2025), this metabolic state is associated with enhanced inflammatory activity (Contreras et al., 2017; Angeli et al., 2021; Kistner et al., 2022), and inflammatory mediators such as IL-6 and TNF-α can impair TJ integrity in epithelial tissues (Al-Sadi et al., 2014; Wu et al., 2025). Although previous studies suggested a link between NEB, metabolic status, and BMB integrity (Lu et al., 2013; Stumpf et al., 2013; Gross et al., 2020), it remains unclear to what extent greater metabolic load alters the closure pattern of the BMB immediately after parturition. Therefore, the objective of this study was to investigate changes of BMB-related markers in cows experiencing different levels of metabolic load after the onset of lactation.

The experimental protocol complied with Swiss law on animal protection and welfare approved by the committee of animal experimentation of the canton of Fribourg (Switzerland, approval numbers 2024-23-FR and 2024-31-FR). The study took place between September and December 2025 and was conducted as part of a larger feeding trial in dairy cows conducted at Agroscope (Posieux, Switzerland). Retrospectively, a subset of 20 multiparous Holstein cows matching the inclusion criteria (no reported dystocia, clinically healthy, and no signs of clinical mastitis) was enrolled. Cows were housed in a tiestall barn and assigned to 2 different feeding regimens starting 42 d antepartum (**a.p.**). The provision of either a high-energy (6.5 MJ NEL/kg DM) or a low-energy (5.0 MJ NEL/kg DM) diet resulted in 2 experimental groups with differing BCS at calving. The high-energy diet was a partial mixed ration (**PMR**; 6. ± 0.1 MJ NEL/kg DM) consisting of hay (38.6% ± 1.3%), grass silage (34.0% ± 0.7%), corn silage (25.0% ± 0.8%), and a protein-rich concentrate (2.4% ± 0.2%, on a DM basis) until parturition. In addition, cows in the high body condition group (**HC**) were supplemented a.p. with 4.7 kg DM/d of an energy-rich concentrate (8.45 MJ NEL/kg of DM). This high-energy feeding regimen led to a high body condition group (HC), consisting of 12 animals with a BCS of 3.77 ± 0.12 (mean values ± SEM) at calving (parity number of 3.08 ± 0.34, range: 2–6). In contrast, the low-energy diet was a PMR (5.0 ± 0.3 MJ NEL/kg DM) composed of straw (32.0% ± 0.0%), hay (26.3% ± 0.9%), grass silage (23.1% ± 0.5%), corn silage (17.0% ± 0.5%), and a protein-rich concentrate (1.6% ± 0.1%). The low-energy feeding regimen resulted in a low-conditioned group (**LC**) consisting of 8 cows with a mean BCS of 2.72 ± 0.12 at calving and parity number 3.13 ± 0.58 (range: 2–5). Fresh feed was provided once daily for ad libitum intake. After calving, the PMR fed to HC cows was offered to LC cows as well. In addition, from calving onward, the LC group received a higher, whereas the HC group received a lower level of energy-rich concentrate supplementation. Supplementation started at 2.3 kg DM/d in the LC group and 0.9 kg DM/d in the HC group and was increased daily thereafter in both experimental groups (additional details on the feeding regimen are provided in Supplemental Table S1, see Notes). This differential p.p. energy supplementation was implemented to further force differences in the metabolic status between experimental groups. Mineral feed was provided according to animal requirements (Agroscope, 2021) and access to fresh water was continuously provided. Shortly before calving, cows were moved to calving pens with deep litter and returned to the tiestall barn thereafter. Individual feed intake was recorded daily by weighing feed refusals before the subsequent feeding. Samples for feed analysis were collected daily. Cows were milked twice daily at 0500 h and 1600 h in a milking parlor with automated milk yield recording. Body weight was automatically measured both after morning and evening milking by a walkover weighing system (Insentec/Hokofarm Group BV, Marknesse, the Netherlands). The BCS was determined at calving according to Edmundson et al. (1989) on a 1 to 5 scale. Colostrum was harvested within 3.1 ± 0.7 h p.p. and at the second milking at 13.1 ± 0.9 h p.p. Milk samples (∼50 mL) from d 1 to 3 p.p. were collected 2 times per day (first 6 consecutive milkings p.p.) and then only during the morning milking from d 4 and 5, and either stored at −20°C until analysis or preserved in bronopol tubes (50 mL, 2-bromo-2-nitropropane-1,3-diol) for analysis of milk gross composition. Blood samples were taken on average 3.0 ± 0.7 h after calving in parallel with colostrum collection and on d 2 to 5 p.p. after the morning milking, between 0530 h and 0800 h. Blood was obtained from the jugular vein into 9-mL K3 EDTA and serum clot activator tubes, put on wet ice, centrifuged at 4°C for 15 min at 1,500 × *g*, further processed as described by Freihofer et al. (2025), and stored at −20°C until analysis. Milk electrolytes (Na, K, Cl), milk albumin (**ALB**), lactate dehydrogenase (**LDH**), and SCC were evaluated as markers of BMB permeability. In addition, milk IgG were detected to further characterize changes in p.p. milk composition. Feed samples were analyzed for DM, ash, OM, NDF, ADF, water-soluble carbohydrates, and CP as described in Eichinger et al. (2025). Milk fat, protein, lactose content, and SCC were determined by mid-infrared spectroscopy (Suisselab AG, Zollikofen, Switzerland), and ECM was calculated as follows: ECM (kg/d) = milk yield (kg/d) × [0.38 × fat (%) + 0.24 × protein (%) + 0.17 × lactose (%)]/3.14 (Agroscope, 2021). The absorbable protein at the duodenum (**APD**) balance was estimated from daily APD intake minus estimated requirements (BW^0.75^ × 3.25 + milk protein yield × 50/32; Agroscope, 2017). Based on average daily BW, daily energy balance (**EB**) was calculated by subtracting energy requirements for maintenance and calculated energy demand for milk production from net energy intake from feed (Agroscope, 2021). Blood plasma concentrations of NEFA (ref. FA115, Randox Laboratories Ltd., United Kingdom) and BHB (ref. RB1007, Randox Laboratories Ltd., United Kingdom), as well as blood serum concentration of ALB (ref. 08056692190, Roche Diagnostics Switzerland) and enzyme activity of LDH (ref. 8057958190, Roche Diagnostics Switzerland) were enzymatically determined using an automated analyzer (Cobas Pure c303, Roche Diagnostics, Switzerland). The same analyzer as for blood was used to determine milk concentrations of ALB (ref. 08056692190), Na (ref. 10825446801), K (ref. 10825441001), Cl (ref. 03246353001), and LDH activity (ref. 8057958190, all Roche Diagnostics Switzerland). Milk immunoglobulin (IgG) concentrations were quantified by ELISA (Bethyl Laboratories Inc.) as described by von Riedheim et al. (2026).

Statistical analysis was performed using R (version 4.4.1, R Studio; https://posit.co/products/open-source/rstudio). Somatic cell count data were log-transformed (log_10_). Longitudinal data were analyzed using a mixed model including experimental group, time, and group × time interaction as fixed effects and cow as a random effect (lme4 package; Bates et al., 2015). For BMB permeability variables, hours p.p. at sampling were included as a nonlinear covariate (second-order polynomial) to account for differences in sampling time p.p. between experimental groups. The significance of fixed effects was evaluated using Type III ANOVA with Satterthwaite's approximation of degrees of freedom (lmerTest package; Kuznetsova et al., 2017). Post hoc pairwise comparisons of estimated marginal means were conducted using Tukey adjustment for multiple testing (package emmeans; Lenth and Piaskowski, 2017). Unless otherwise stated, data are presented as mean values ± SEM. Statistical significance was declared at *P* < 0.05, and trends were assumed at 0.05 ≤ *P* < 0.10.

Dry matter intake did not differ between experimental groups (*P* = 0.36) and increased over time (*P* < 0.001), whereby DMI increased more slowly in the HC group (*P* = 0.02; Table 1). Body weight shortly before calving was higher in HC cows (854 ± 24 kg) than LC cows (724 ± 23 kg; *P* = 0.002; data not shown). Milk fat (HC 5.4% ± 0.2% vs. LC 4.5% ± 0.2%; *P* = 0.19), milk protein (HC 6.8% ± 0.4% vs. LC 7.0% ± 0.5%; *P* = 0.97), and lactose content (HC 4.0% ± 0.1% vs. LC 4.1% ± 0.1%; *P* = 0.33) did not differ between experimental groups. Milk yield and ECM yield were greater in HC cows than LC cows (*P* < 0.05 and *P* < 0.01) and increased over time (*P* < 0.001; Table 1). The HC cows experienced a more pronounced negative EB and APD balance during the first 5 d p.p. compared with LC cows (−50.4 ± 5.5 MJ/d vs. −6.7 ± 4.7 MJ/d; *P* < 0.01 and −989.2 ± 55.4 g/d vs. −462.7 ± 112.4 g/d; *P* = 0.011) and tended to show a steeper decline in EB over time (*P* = 0.09; Table 1). The NEFA concentrations differed between experimental groups (*P* < 0.01) and increased over time (*P* = 0.03), especially from d 2 p.p. onward, where NEFA concentrations increased in HC cows while remaining lower in LC cows (0.63 ± 0.06 mmol/L vs. 0.21 ± 0.03 mmol/L; group × time; *P* = 0.04; Table 1). The BHB concentrations were greater in HC cows (0.70 ± 0.02 mmol/L vs. 0.55 ± 0.02 mmol/L; *P* < 0.01) and increased over time (*P* = 0.03) with no significant interaction (*P* = 0.24; Table 1). No differences between experimental groups were observed for ALB concentrations (HC 40.2 ± 0.3 g/L vs. LC 38.9 ± 0.4 g/L; *P* = 0.24; data not shown) or LDH activity in blood (HC 1,081 ± 27 U/L vs. LC 1,082 ± 33 U/L; *P* = 0.92; data not shown) or in milk (*P* = 0.79 and *P* = 0.60, respectively; Figure 1A and 1B). However, all 4 parameters (serum ALB and LDH; milk ALB and LDH) were affected by time (all *P* < 0.05), with serum LDH increasing, serum ALB increasing after d 2 p.p., and milk ALB and LDH concentrations decreasing over time. Total IgG concentrations in milk did not differ between experimental groups (HC 18.7 ± 3.0 g/L vs. LC 17.2 ± 3.5 g/L; *P* = 0.51), and no group-time interaction was observed (*P* = 0.85), whereby IgG concentrations decreased over time (*P* < 0.001; data not shown). Somatic cell count did not differ between experimental groups (*P* = 0.80) but decreased over time (*P* < 0.01; Figure 1C). Milk concentrations of Na and K tended to be greater in HC cows and lower in LC cows (both *P* = 0.09; Figure 2A and B, respectively), whereas milk Cl concentrations did not differ between experimental groups (*P* = 0.86; Figure 2 C). Sodium concentrations decreased rapidly until d 2 p.p., followed by a slower decrease until d 5 (*P* < 0.001), whereas K concentrations did not change over time (*P* = 0.16). Chloride concentrations decreased from the first milking to the morning milking on d 5 in both experimental groups (*P* < 0.001), with more pronounced fluctuations in LC than in HC cows (*P* < 0.01). Greater milk chloride concentrations in the HC group were only recorded in the second milking (*P* = 0.02). The Na/K ratio tended to be greater in HC than LC cows (HC 0.55 ± 0.01 vs. LC 0.48 ± 0.01; *P* = 0.09) and decreased until d 2 p.p., stabilizing thereafter (time *P* < 0.001; group × time *P* = 0.11; data not shown). The present study evaluated changes of BMB integrity-related parameters dynamics in Holstein dairy cows differing in metabolic status at the onset of lactation. Physiologically, mammary epithelial permeability is transiently increased around parturition and declines during the first milkings as TJ close and a functional secretory epithelium is established (Wellnitz and Bruckmaier, 2021). We tested the hypothesis whether a more pronounced metabolic load at the onset of lactation alters the postpartal BMB closure pattern. The experimental model successfully induced distinct metabolic conditions during the first week of lactation. Dry matter intake did not differ between experimental groups in the present study despite different BCS at calving. However, HC cows showed greater milk and ECM yields than LC cows and, under the more energy restricted p.p. feeding regimen, experienced a more severe NEB compared with LC cows. This was accompanied by greater NEFA and BHB concentrations in HC cows, indicating enhanced adipose tissue mobilization and ketogenesis (Wankhade et al., 2017) and thus a greater degree of metabolic load in HC than in LC cows (Toralová et al., 2025). In both humans and dairy cows, metabolic stress has been associated with increased inflammatory activity, including elevated concentrations of cytokines such as TNF-α and IL-6 (Hotamisligil, 2017; Wnorowska et al., 2025). In human epithelia, IL-6 has been shown to impair barrier integrity by remodeling TJ and increasing paracellular permeability (Al-Sadi et al., 2014). Whether similar mechanisms contribute to changes in bovine mammary BMB integrity remains unclear, although elevated IL-6 concentrations have been reported in the mammary tissue of cows with ketosis-associated metabolic stress (Sun et al., 2021). Because inflammatory cytokines were not measured in the present study, this pathway remains speculative. Nevertheless, based on the available literature, we hypothesized that a greater metabolic load may alter BMB permeability after calving. To assess this, we quantified several markers of BMB permeability. In line with Wall et al. (2015), albumin and LDH activity were used as markers of blood-derived components transferred into milk. Consistent with previous studies, these parameters (ALB, LDH) showed the expected rapid p.p. decline, reflecting the physiological BMB closure during the first days of lactation (Wall et al., 2015; Kessler et al., 2019). Thus, the overall temporal patterns observed in the present study were consistent with previous findings on the postpartum BMB remodeling. Despite marked differences in metabolic load between HC and LC cows, the protein-based indicators of BMB permeability (milk ALB, milk LDH, and SCC) did not reveal any differences between experimental groups, suggesting that the overall course of p.p. BMB remodeling was largely preserved and not affected by the accompanying metabolic status. Thus, despite clear differences in metabolic load between HC and LC cows, the temporal patterns of these markers substantially overlapped. In contrast, milk electrolytes revealed transient group-related differences during the first milkings. Colostrum is characterized by elevated Na and Cl concentrations and low K concentrations. This pattern reverses as TJ close during the transition to mature milk during the first milkings (Nguyen and Neville, 1998). Consequently, milk Na, Cl, and K concentrations, as well as the Na/K ratio, are used as indirect indicators of TJ permeability (Stelwagen et al., 1999; Sorensen et al., 2001). In the present study, greater Na and Cl concentrations during the first 2 milkings, lower K concentrations at the onset of lactation, and a tendency for greater Na concentrations, a tendency toward lower K concentrations and tendency for greater Na/K ratio in the HC indicate that the BMB permeability in this group was greater at the onset of lactation compared with LC cows. The discrepancy between protein-based and electrolyte-based markers raises the question whether these parameters are equally sensitive in describing BMB permeability. This is particularly relevant for milk ALB, as ALB is known to bind to the FcRn receptor, which mediates the transcellular transport of IgG1, yet evidence for FcRn-driven ALB transport into milk remains scarce (Baumrucker et al., 2021). Because BMB closure is a gradual rather than a binary process, the decrease in markers of paracellular transport through TJ is not only time dependent but also depends on physicochemical properties such as molecular size (Wellnitz and Bruckmaier, 2021; Stelwagen, 2025). In this context, Wall et al. (2015) suggested that prolonged detection of α-lactalbumin compared with LDH or ALB may reflect size-dependent passage across the BMB. The absence of group differences in protein-based BMB permeability markers in the present study, despite detected differences in Na, K, Cl, and the Na/K ratio, therefore suggest that smaller electrolytes may capture subtle alterations in epithelial permeability more sensitively than larger blood-derived proteins. Notably, even electrolyte group differences were restricted to the first 2 milkings, precisely when BMB remodeling is most dynamic and the largest differences between groups would be expected. The fact that these differences did not persist beyond this narrow time window suggests a small overall effect of metabolic load on BMB permeability in this study. Nevertheless, this explanation remains speculative and should be interpreted with caution, as a direct qualitative or quantitative comparison of the sensitivity of different BMB permeability markers is hardly possible in the present study, particularly because plasma α-lactalbumin was not measured due to limited sample volume. The present findings partly contrast with earlier reports linking metabolic status to impaired BMB function. For instance, Gross et al. (2020) reported that cows with a more severe NEB and subsequent greater BHB concentrations exhibited a delayed recovery of BMB integrity following an acute inflammatory challenge, as indicated by prolonged elevations of milk SCC, LDH, IgG, and ALB.

**Table 1:** Least squares means (±SEM) of performance and metabolic status variables during the first 5 d postpartum in multiparous Holstein cows (n = 20) fed either a high-energy (HC, n = 12) or low-energy (LC, n = 8) diet antepartum and differential postpartum concentrate supplementation to amplify metabolic divergence

Item	High-condition group (BCS: 3.77 ± 0.12)	Low-condition group (BCS: 2.72 ± 0.12)	*P*-value group^1^	*P*-value time^1^	*P-*value group × time^1^
Total DMI (kg/d)	16.2 ± 0.4	17.2 ± 0.5	0.357	<0.001	0.022
Milk yield (kg/d)	23.9 ± 1.1	19.2 ± 1.2	0.032	<0.001	0.604
ECM (kg/d)	32.7 ± 1.7	23.2 ± 1.3	0.008	<0.001	0.787
EB (MJ)	−50.4 ± 5.5	−6.7 ± 4.7	0.002	0.003	0.088
APD balance^2^ (g/d)	−989.2 ± 554.5	−462.7 ± 112.4	0.011	0.003	0.859
NEFA (mmol/L)	0.63 ± 0.06	0.21 ± 0.03	0.002	0.025	0.035
BHB (mmol/L)	0.70 ± 0.02	0.55 ± 0.02	0.001	0.026	0.240

1 *P*-values were obtained by Type III ANOVA from a linear mixed model with group, time, and group × time as fixed effects and animal as a random effect.

2 APD = absorbable protein in the duodenum. The APD balance was estimated from daily APD intake minus estimated requirements (BW^0.75^ × 3.25 + milk protein yield × 50/32; Agroscope, 2017).

**Figure 1:**
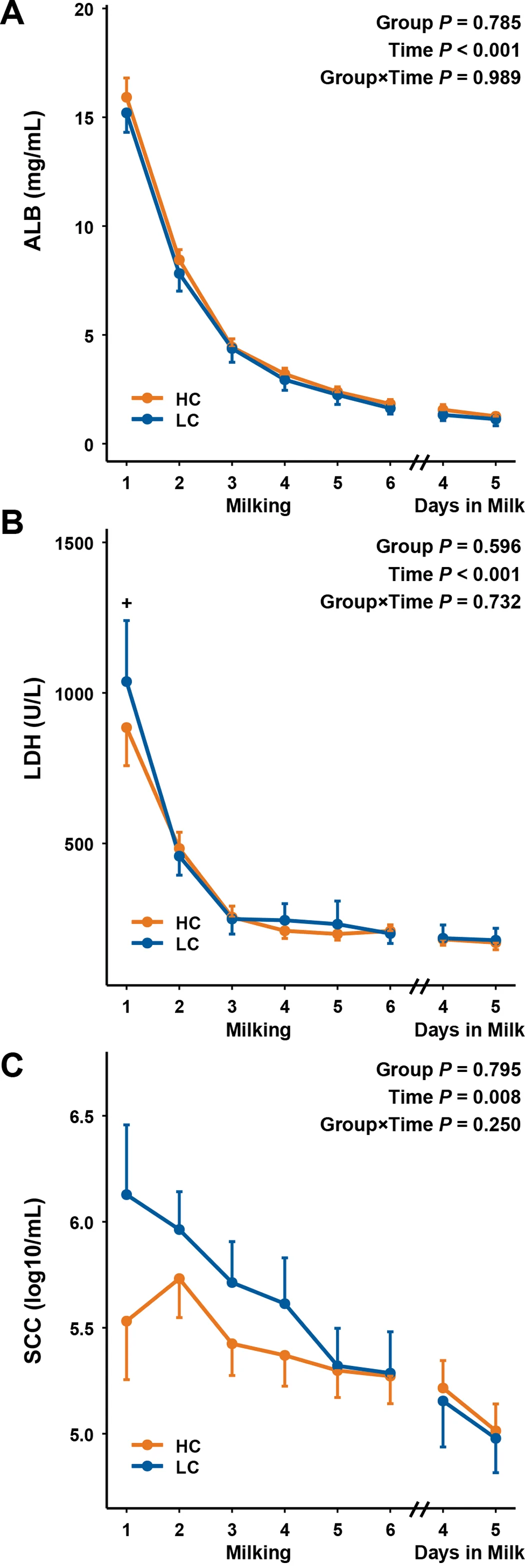
Milk concentration of albumin (ALB; A), activity of lactate dehydrogenase (LDH; B), and SCC (C) in high (HC) and low condition (LC) cows at the first 6 consecutive milkings after parturition and on d 4 to 5 of lactation. Data presented are mean values ± SEM. Differences between experimental groups at the same time point are marked by + (*P* < 0.10).

**Figure 2:**
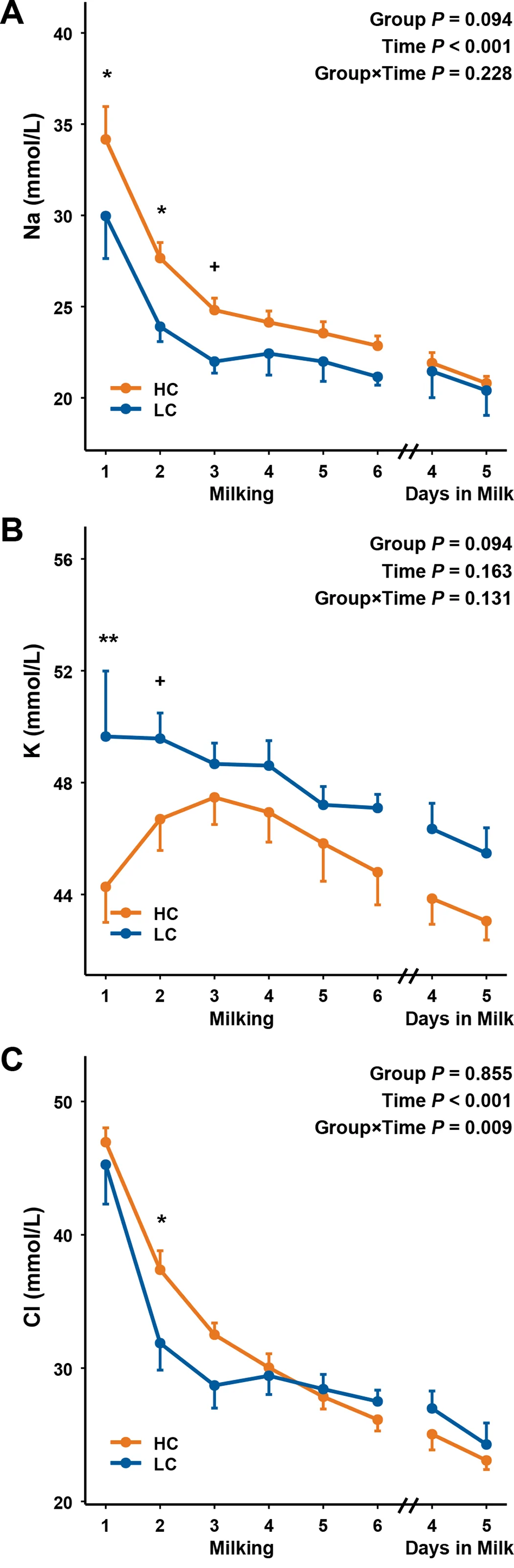
Milk concentrations of sodium (A), potassium (B), and chloride (C) in high- (HC) and low-condition (LC) cows at the first 6 consecutive milkings after parturition and on d 4 to 5 of lactation. Data presented are mean values ± SEM. Differences between experimental groups at the same time point are marked by **P < 0.01, *P < 0.05, or +*P* < 0.10.

In conclusion, an elevated metabolic load at the onset of lactation had, at most, a modest effect on BMB permeability dynamics. Whereas protein-based markers and SCC did not support a major delay in p.p. BMB integrity, electrolyte alterations suggest that a mild and transient increase in permeability in HC cows cannot be excluded. However, further research is warranted to clarify this aspect. Based on the present findings, the development of a functional BMB after calving appears to be largely unaffected by metabolic stress.
